# Emergence of clonal chromosomal alterations during the mesenchymal stromal cell cultivation

**DOI:** 10.1186/s13039-015-0197-5

**Published:** 2015-12-01

**Authors:** Tamara Borgonovo, Maria Marlene Solarewicz, Isadora May Vaz, Debora Daga, Carmen Lúcia Kuniyoshi Rebelatto, Alexandra Cristina Senegaglia, Enilze Ribeiro, Iglenir João Cavalli, Paulo Slud Brofman

**Affiliations:** Centro de Tecnologia Celular (CTC), Pontifícia Universidade Católica do Paraná (PUCPR), Curitiba, Paraná Brazil; Departamento de Genética, Universidade Federal do Paraná (UFPR), Curitiba, Paraná Brazil; Cytogenetic Laboratory of Centro de Tecnololgia Celular (CTC), PUCPR, Curitiba, Brazil

**Keywords:** Mesenchymal stromal cells, Karyotype, Genomic integrity, Regenerative medicine

## Abstract

**Background:**

Use of human mesenchymal stromal cells (MSCs) is a promising strategy for cell therapy in injured tissues recovery. However, MSCs acquire genetic changes when cultivated *in vitro* that make them more susceptible to undergo neoplastic transformation. Therefore, genomic integrity of stem cells should be monitored carefully for the use in basic research and clinical trials, including karyotype analysis to confirm the absence of genetic instability. Here, we report a case of a male 67-year-old patient selected to join the study: “Autologous transplantation of mesenchymal cells for treatment of severe and refractory ischemic cardiomyopathy”. He underwent nephrectomy for malignant tumor on the right kidney. Cytogenetic analysis on a bone marrow sample showed a normal karyotype: 46,XY[20]. However, the MSC at second passage showed a hyperdiploid clone, with clonal trisomies of chromosomes 4, 5, 10 and X. In order to investigate more, another sample from the same patient was used for a second cultivation and, at third passage, these cells showed a clonal translocation t(9;18)(p24;q11). The recurrent aberrations in MSC may indicate the beginning of a spontaneous transformation in culture, so, these cells were not used for cell therapy. Several analyses were performed at the Center for Cell Technology (152 samples), however this was the only case to show clonal cytogenetic abnormalities. Interestingly, two distinct clonal alterations were seen in two parallel cell cultivations from the same patient, suggesting a propensity for genetic instability. This highlights the need to evaluate these cells on a case-by-case basis, especially in patients with a history of cancer. Although there is controversy about the use of cells with cytogenetic abnormality for therapy, because their tumorigenic doubtful potential, we decided against the use of these cells for regenerative medicine.

**Case presentation:**

Here, we report a case of a male 67-year-old patient selected to join the study: “Autologous transplantation of mesenchymal cells for treatment of severe and refractory ischemic cardiomyopathy”. He underwent nephrectomy for malignant tumor on the right kidney. Cytogenetic analysis on a bone marrow sample showed a normal karyotype: 46,XY[20]. However, the MSC at second passage showed a hyperdiploid clone, with clonal trisomies of chromosomes 4, 5, 10 and X. In order to investigate more, another sample from the same patient was used for a second cultivation and, at third passage, these cells showed a clonal translocation t(9;18)(p24;q11). The recurrent aberrations in MSC may indicate the beginning of a spontaneous transformation in culture, so, these cells were not used for cell therapy. Several analyses were performed at the Center for Cell Technology(152 samples), however this was the only case to show clonal cytogenetic abnormalities. Interestingly, two distinct clonal alterations were seen in two parallel cell cultivations from the same patient, suggesting a propensity for genetic instability. This highlights the need to evaluate these cells on a case-by-case basis, especially in patients with a history of cancer.

**Conclusions:**

Although there is controversy about the use of cells with cytogenetic abnormality for therapy, because their tumorigenic doubtful potential, we decided against the use of these cells forregenerative medicine.

## Background

Use of human mesenchymal stromal cells (MSCs) is a promising strategy for cell therapy in injured tissues recovery. However, MSCs acquire genetic changes when cultivated *in vitro* that make them more susceptible to undergo neoplastic transformation. Therefore, genomic integrity of stem cells should be monitored carefully for use in basic research and clinical trials [1]. MSCs, like products of advanced therapy, should satisfy all the requirements for human use of medicinal products, including karyotype analysis to confirm the absence of genetic instability, to ensure their quality and safety [2]. At the Center for Cell Technology (CTC-PUCPR), we regularly perform cytogenetic analyses (G-band karyotyping) for quality control of the cells.

## Case presentation

Here, we report the case of a 67-year-old patient, male, who underwent coronary artery bypass grafting at 54 years of age after a diagnosis of myocardial infarction. An angioplasty and 13 coronary angiography tests were performed. In 1992, he underwent nephrectomy for a malignant tumor in the right kidney. Scintigraphy performed in 2014 showed moderate and transient hypocaptation (ischemia) in the anteroseptal middle and basal region of the left ventricle. The patient was then enrolled in the study “Autologous transplantation of mesenchymal cells for treatment of severe and refractory ischemic cardiomyopathy” (Local Ethics Committee number: 5250). His bone marrow mononuclear cells were collected and isolated by performing density gradient centrifugation and were used as a source of MSCs. These cells were loaded onto Histopaque® (1.077 g/mL; Sigma Chemical Co., St. Louis, MO) and were seeded in culture flasks at a density of 2 × 10^6^ cells/cm^2^. MSCs were cultivated in Iscove’s modified Dulbecco’s medium (Gibco™) supplemented with 15 % fetal bovine serum (Gibco™), in a humidified incubator at 37 °C with an atmosphere of 5 % carbon dioxide. After the adhesion of MSCs to the flasks, their culture medium was changed every 3–4 days. After reaching approximately 80 % confluence, the MSCs were dissociated using 0.25 % trypsin–ethylenediaminetetraacetic acid (EDTA—Sigma–Aldrich) and were continually expanded at least until the second passage (P2). These cells were evaluated for performing cytogenetic studies; immunophenotyping; and osteogenic, adipogenic, and chondrogenic differentiation assays [3, 4], according to the minimal step of the standard criteria established by the International Society for Cellular Therapy [5].

**Fig. 1:**
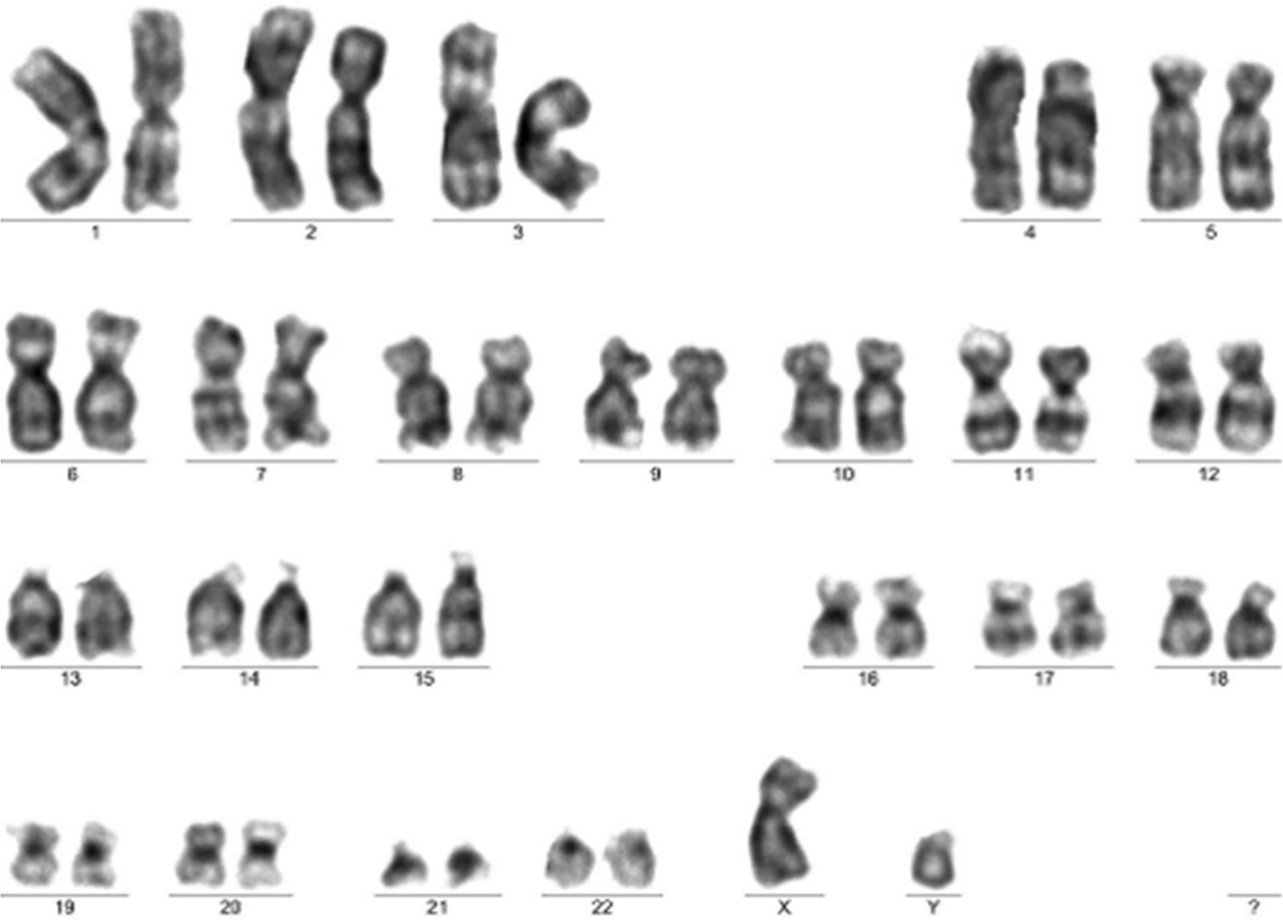
Normal karyotype. Karyogram before cell expansion (bone marrow cells). Karyotype: 46,XY

Cytogenetic analyses were performed before and after cell expansion. Standard cytogenetic procedures were used to perform the cytogenetic analysis of the bone marrow sample. This patient showed a normal karyotype: 46,XY[20] (Fig. 1).

**Fig. 2:**
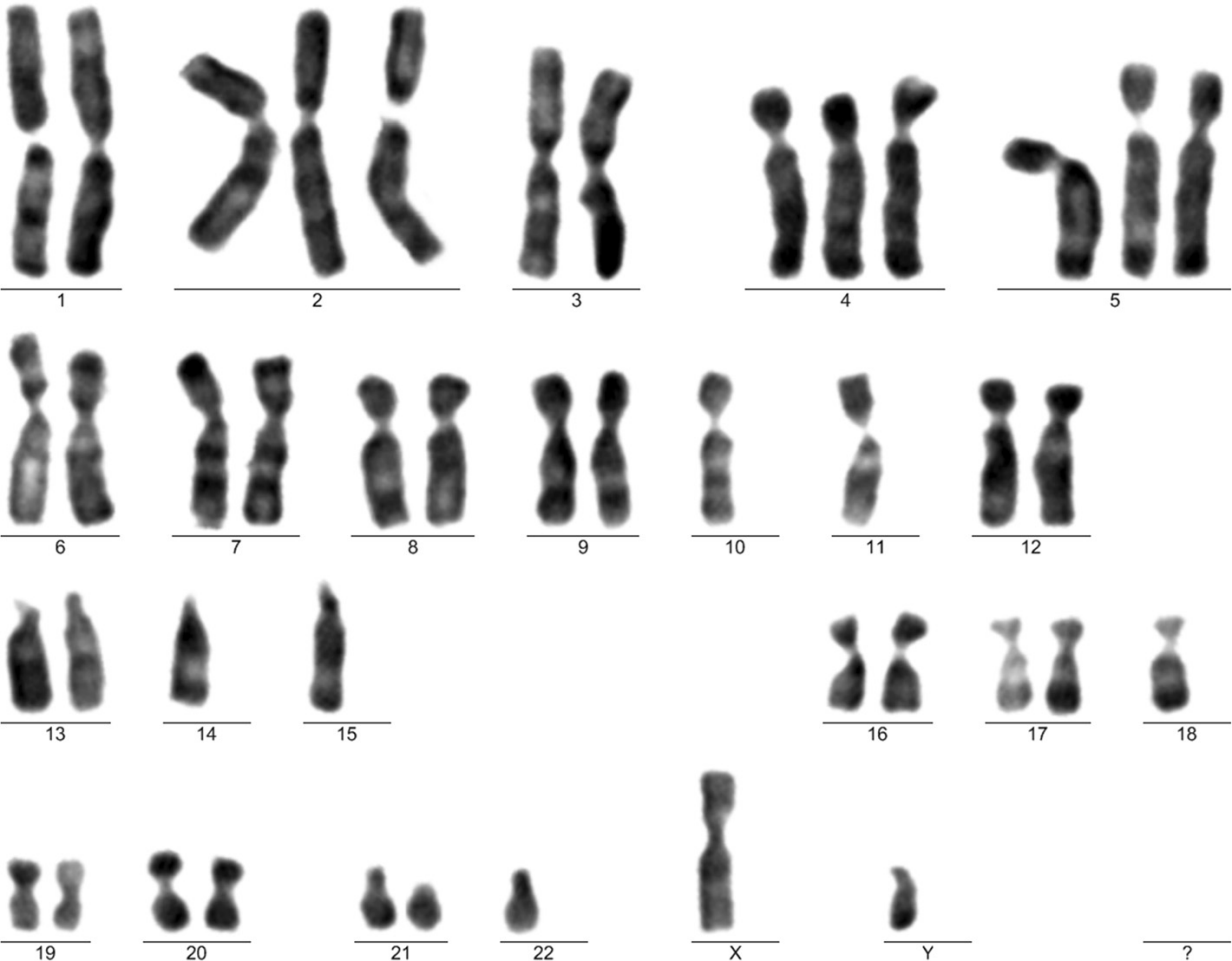
Karyotype with trisomies. Karyogram after cell expansion, from the first cultivation (mesenchymal stem cells at second passage). Karyotype: 43,XY, +2,+4,+5,−10,−11,−14,−15,−18,−22

MSCs were evaluated using a protocol described in our previous study [6]. Five MSC samples obtained from different passages (P) were analyzed. In the first cultivation, the MSC at second passage (P2) showed a hyperdiploid clone (Fig. 2), with a composite karyotype: 43 ~ 52,XY,+X,+4,+5,+10[cp4]/46,XY[18], and non-clonal trisomies:+2,+12,+20.

This sample was not approved for infusion according to the criteria established by the CTC-PUCPR and those published previously [7, 8]. After that, the MSCs from P4 and P5 showed a normal karyotype, 46,XY[26] and 44 ~ 46,XY[13], respectively.

**Fig. 3:**
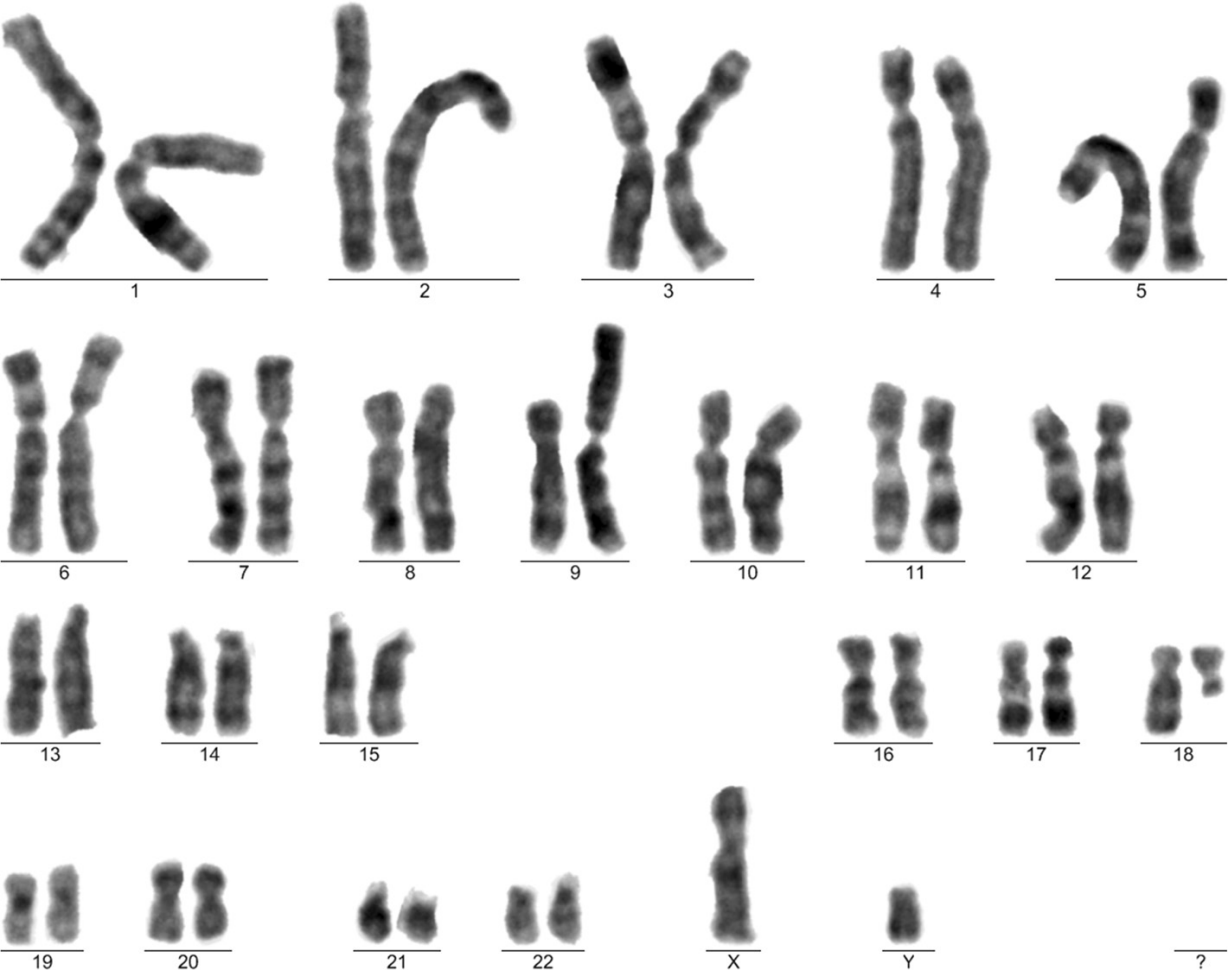
Karyotype with translocation. Karyogram after cell expansion, from the second cultivation (mesenchymal stem cells at third passage). Karyotype: 46,XY, t(9;18)(p24;q11)

In order to investigate more, another sample of mononuclear cells (that had been frozen for backup) from the same patient, was used for a second cultivation. At P3, these cells showed a clonal translocation: 46,XY,t(9;18)(p24;q11)[8]/46,XY[5] (Fig. 3). At P5 the karyotype was 26 ~ 44,XY[10].

Out of all cytogenetic analyses performed at the CTC-PUCPR (152 samples), this was the only case to show clonal cytogenetic abnormalities. Therefore, these cells were not used for cell therapy.

Different researchers have different views regarding the use of cells with cytogenetic abnormalities for cell therapy because of their tumorigenic doubtful potential.

Karyotype abnormalities are frequently observed in pluripotent cells, but also occur in adult stem cells. This has caused discussion between different groups about the risk factors for tumorigenic potential [9]. Sensebé et al. [10] suggested that genomic stability observed in MSC is robust and that there was no significant risk of neoplastic transformation. They stated that the presence of acquired chromosomal aberrations in the first moment, followed by a normal karyotype, suggested that this aberration did not confer any growth advantage.

In response to the above suggestion, Uri Ben David [11] argued that there are two types of genomic aberrations: (1) transient aberrations, which occasionally appear in culture but are disadvantageous and hence disappeared during propagation, and (2) advantageous recurrent aberrations, which rapidly accumulate in the culture in a clonal manner. These two types of genomic aberrations exist simultaneously in stem cell cultures. Sensebé discussed the former type of aberrations. However, it should be noted that aberrant cells can outgrow normal cells. Therefore, clonal aberrations in MSCs may indicate the initiation of a spontaneous transformation in a culture. In addition, each stem cell type can acquire distinct recurrent chromosomal abnormalities. Importantly, the common aberrations in stem cell cultures resemble characteristic aberrations in tumors cells from same cell lineages [11].

Both the types of aberrations identified in our patient have been described in several types of cancers (http://cgap.nci.nih.gov/Chromosomes/RecurrentAberrations) [12]. Hyperdiploid karyotypes, including trisomies detected in this case, are common in leukemias and solid tumors. Translocations involving 18q11 have been described in several cases of leukemia and sarcomas, of mesenchymal origin.

It is important to note that aberrations identified in this study, i.e., trisomies (aneuploidies) and translocation (structural), were clonal. They were determined by G-band karyotyping, which is the gold standard technique for detecting clonal abnormalities (both numeric and structural) in cultured cells. This is important because recurrence of clonal chromosomal aberrations is a characteristic of cancers.

Therefore, Barkholt [7] suggested performing a karyotyping analysis in order to exclude products containing cells with abnormalities (which potentially confer a proliferative advantage) according to criteria mentioned in the “Definition of a clone” established by the International System for Human Cytogenetic Nomenclature (2013) [13].

Interestingly, two distinct clonal alterations were seen in two parallel cell cultivations from the same patient, suggesting a propensity for genetic instability probably associated with the cancer previous history of this patient. This highlights the need to evaluate these cells on a case-by-case basis, especially in patients with a history of cancer. In this case, separated runs in culture resulted in the emergence of different end products (different clonal chromosome aberrations). This is evidence of the population’s genome diversity, which is increased by chaotic chromosome changes, during the chromosome instability observed in genome evolution of tumor cells. In this process, there is a phase, referred to as punctuated/discontinuous, and it is characterized by the presence of non-clonal aberrations and transitional clonal aberrations [14], as we observed on two cultivations.

On another hand, the emergence of aneuploidies (changes in chromosome number) during cell culture is common, and some studies have shown that this is not necessarily associated with tumor formation in immunodeficient mice [15]. However, Barkholt [7] stated that even though transformed cells do not seem to undergo tumor formation, this risk should be considered because the characteristics of these abnormal cells are uncertain and because the follow-up of patients is limited.

## Conclusion

In practice, the decision to infuse cells with abnormal karyotypes should be taken within a few hours. If an aberration is detected in cells from P2 when a patient is about to receive these cells, there is no time to wait for the results of cytogenetic analyses of cells from subsequent passages to determine whether this aberration is temporary. There are no lists available in the literature about recurrent aberrations in MSCs—as there is for neoplastic cells [12]—nor of which aberration would be transitory and which ones would be potentially tumorigenic. Therefore, we decided, similar to that suggested by Muntion et al. [16], against the use of these cells for regenerative medicine.

## Consent

Written informed consent was obtained from the patient for publication of this Case report and any accompanying images. A copy of the written consent is available for review by the Editor-in-Chief of this journal.

## Abbreviations

MSC: Mesenchymal stromal cells
CTC-PUCPR: Center for Cell Technology of Pontifical University Catholic of Paraná
P: Passage
